# Nintedanib-Induced Colitis Treated Effectively With Budesonide

**DOI:** 10.7759/cureus.9489

**Published:** 2020-07-31

**Authors:** Afshin Amini, Elliott Koury, Elie Chahla

**Affiliations:** 1 Internal Medicine, St. Luke's Hospital, Chesterfield, USA; 2 Internal Medicine/Gastroenterology and Hepatology, St. Luke's Hospital, Chesterfield, USA

**Keywords:** budesonide, nintedanib induced colitis

## Abstract

A 68-year-old male with a past medical history of interstitial pulmonary fibrosis (IPF) on nintedanib and chronic nintedanib-induced diarrhea for three years presented with hematochezia and worsening diarrhea. Diarrhea had persisted despite the use of cholestyramine and oral antidiarrhea medications. As part of the evaluation of diarrhea, he had undergone colonoscopy two years prior, which had shown non-specific moderate diffuse colitis. No significant abnormalities had been noted on physical exam and lab tests. On the present admission, colonoscopy showed diffuse erythematous, friable, and granular mucosa throughout the entire colon. Biopsies were taken and pathology was reported as acute superficial inflammation and possible nintedanib-induced colitis. Since the patient wanted to continue nintedanib as a part of IPF treatment, 9 mg oral budesonide was started, and the patient was followed up after four months. At his follow-up visit, the patient reported that diarrhea had completely resolved.

In this report, we illustrate and discuss a case of nintedanib-induced colitis, which can be resistant to oral antidiarrhea medication and cholestyramine. The mechanism of this side effect is not completely understood; however, it may be related to direct inflammation of the intestinal epithelium, given that nintedanib metabolites are excreted primarily in the stool. As a result, it has been hypothesized that steroids could potentially treat this diarrhea by relieving this inflammation. In our patient, we elected to use budesonide due to less associated systemic side effects and possible similarity of inflammation between nintedanib-associated colitis and inflammatory bowel disease.

## Introduction

Nintedanib, a tyrosine kinase inhibitor, displays antifibrotic activity via blockade of three receptors [platelet-derived growth factor receptor (PDGFR), vascular endothelial growth factor receptor (VEGFR), and fibroblast growth factor receptor (FGFR)] [1]. This drug was initially developed as an anti-tumor agent but was later recognized for its unique antifibrotic activity [2]. It is mainly cleared by liver metabolism, with most of the metabolites being excreted in the feces (feces: 93.4%, urine: <1%) [3]. The most common adverse effect associated with nintedanib is diarrhea (62%), which has led to a permanent dose reduction in 11% of patients and discontinuation in 5% [4]. In this report, we discuss a case of nintedanib-induced diarrhea with complete clinical resolution after treatment with oral budesonide.

## Case presentation

A 68-year-old male with a past medical history of interstitial pulmonary fibrosis (IPF) and chronic diarrhea for three years was admitted to the hospital with the chief complaints of hematochezia and worsening diarrhea. He denied any abdominal pain or nausea. In the past three years, he had been taking nintedanib (150 mg twice daily) for IPF. For his diarrhea, he had been on cholestyramine twice a day and other antidiarrheals, but his diarrhea had persisted and worsened. A colonoscopy performed two years ago had shown non-specific moderate diffuse colitis.

**Figure 1:**
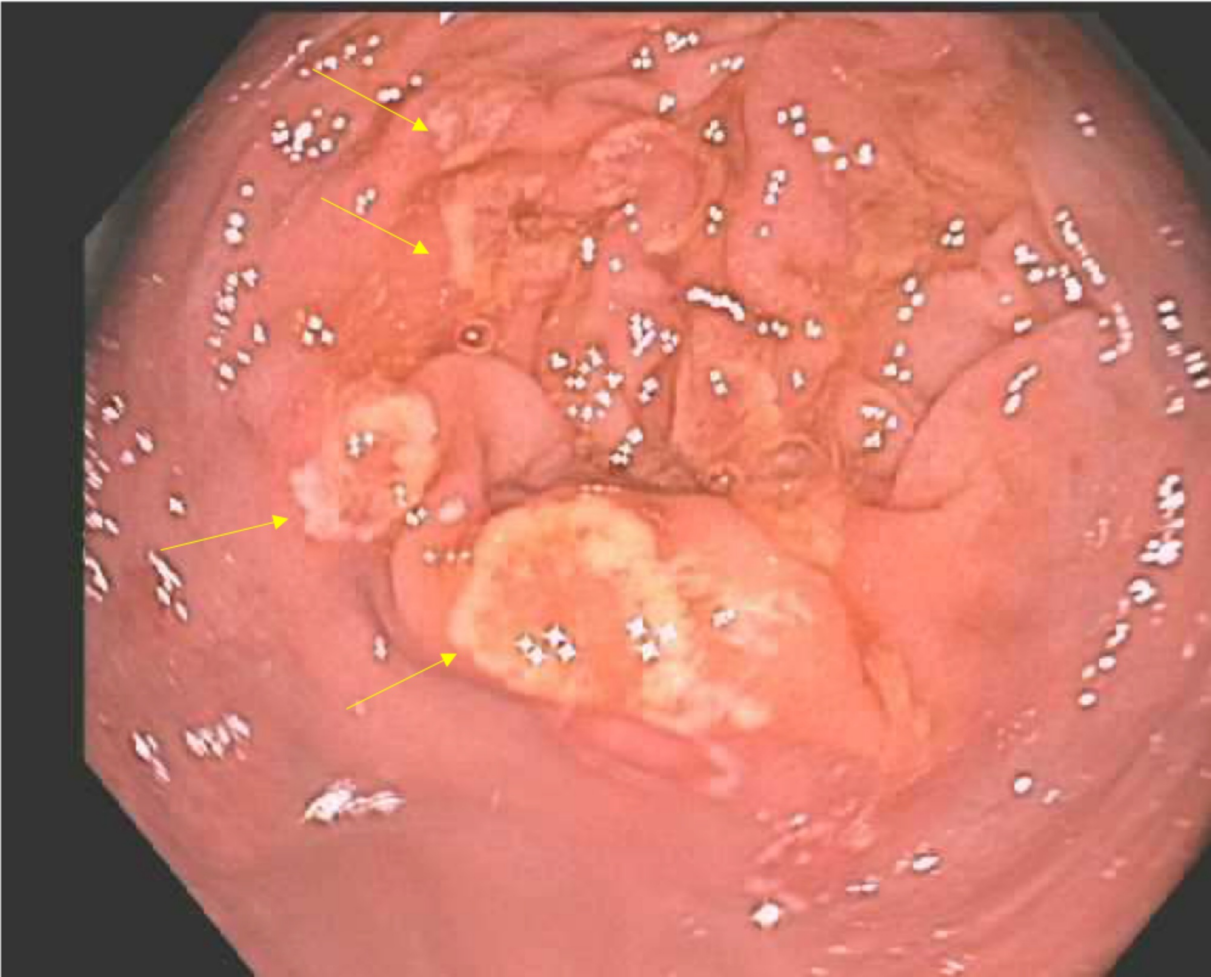
Colonoscopy view The image shows erythematous, friable, and granular mucosa in the cecum (yellow arrows)

**Figure 2:**
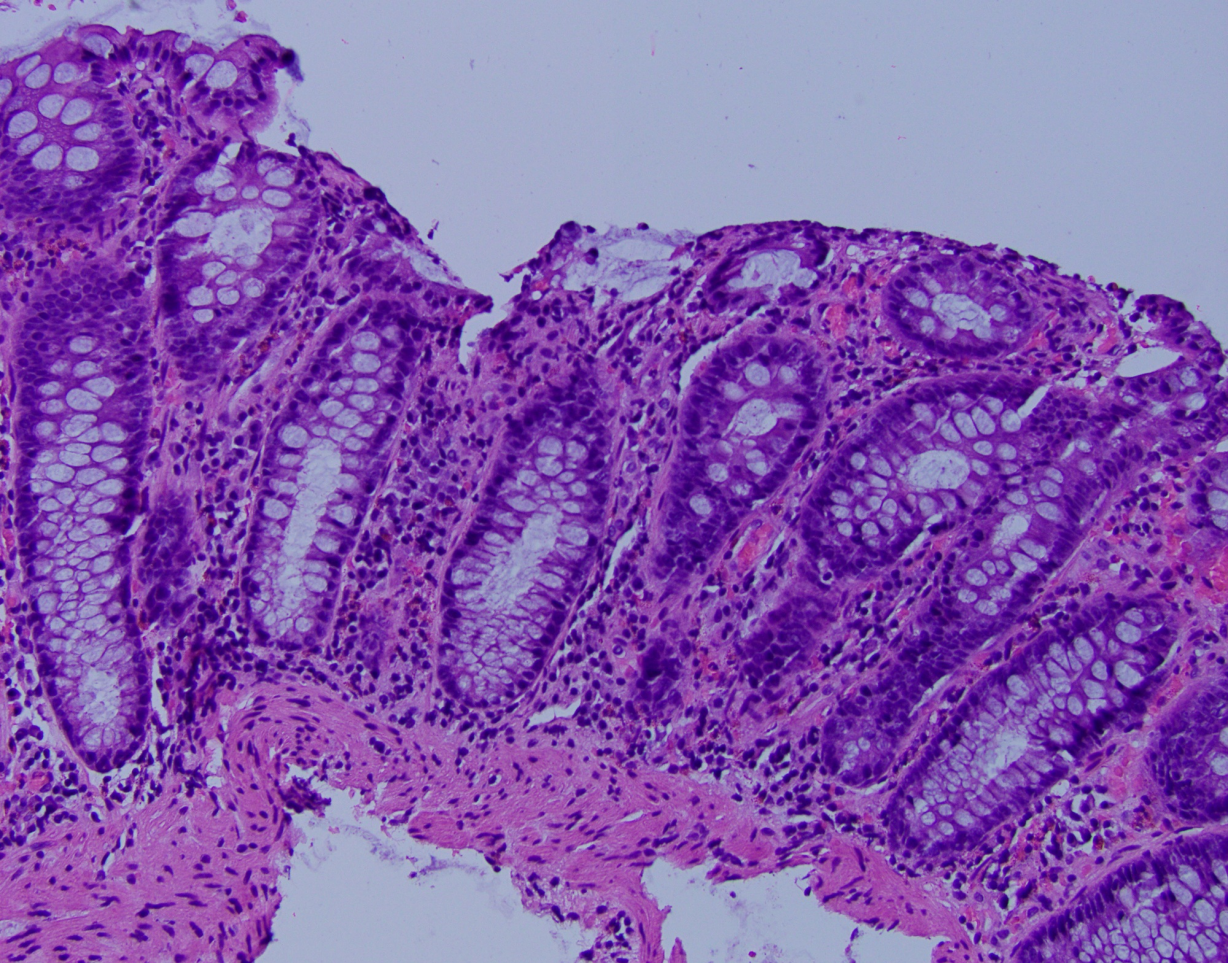
Histopathology of colon biopsy (200x) The image shows acute superficial inflammation, and expansion of lamina propria by lymphoplasmacytic infiltrate

The physical examination and vital signs were unremarkable. His blood work, including complete blood count (CBC) and comprehensive metabolic panel (CMP), was within normal limits. His C-reactive protein (CRP) was mildly elevated. Repeat colonoscopy revealed diffuse areas of erythematous, friable, and granular mucosa throughout the entire colon, similar to the previous endoscopic findings (Figure 1). Histopathology showed acute superficial inflammation, and expansion of lamina propria by lymphoplasmacytic infiltrate, raising the possibility of nintedanib-induced colitis (Figure 2).

As it was more pertinent to continue with nintedanib for his IPF, we elected to treat his colitis with budesonide. He was started on 9 mg oral budesonide with the plan to slowly taper it to the minimum effective dose. His diarrhea gradually improved, and at his follow-up visit about four months later, it had completely resolved.

## Discussion

Nintedanib is an effective treatment for IPF and is associated with reduced disease progression. The use of nintedanib in pulmonary fibrosis was evaluated in the “Efficacy and Safety of Nintedanib in Idiopathic Pulmonary Fibrosis” (INPULSIS-1 and INPULSIS-2) clinical trials [4]. These studies evaluated the safety and efficacy of 150 mg of nintedanib twice daily compared with placebo in patients with idiopathic pulmonary fibrosis. Diarrhea was the most frequently reported adverse event in the nintedanib groups in both trials. In both trials, diarrhea was seen in more than 60% of the patients taking nintedanib, as compared to 18% of patients in the placebo group. Diarrhea led to permanent dose reduction in 11% of patients and discontinuation in 5% [4].

In the “To Improve Pulmonary Fibrosis with BIBF 1120” (TOMORROW) study, a total of 432 patients underwent randomization to receive nintedanib at one of four doses (50 mg once a day, 50 mg twice a day, 100 mg twice a day, or 150 mg twice a day) or placebo for 12 months [5]. The adverse events most frequently leading to discontinuation were diarrhea, nausea, and vomiting. In the group receiving 150 mg twice a day, the rate of diarrhea was 11.8% as compared to 0% in the placebo group. Among the 85 patients in this high-dose group, 47 (55.3%) had diarrhea compared to 15.3% in the placebo group. Four (4.7%) patients had severe diarrhea and three (3.5%) had what was described as serious diarrhea, as compared with zero patients in the placebo group [5].

Kato et al. studied 77 patients with IPF who received nintedanib and showed that 27 patients (35.1%) developed diarrhea that was grade 2 or more severe [6]. Among these, 10 patients required discontinuation of nintedanib despite the use of antidiarrheal medications. Additionally, they reported that the use of concomitant prednisolone successfully prevented diarrhea in patients on nintedanib [6].

The mechanism of nintedanib-induced diarrhea/colitis remains unknown. One of the proposed mechanism involves direct inflammation of the intestinal epithelium induced by nintedanib decomposition products. Nintedanib is primarily cleared via liver metabolism, with most of the metabolites being excreted in the feces [3]. This inflammation, like that of inflammatory bowel disease, may respond to corticosteroid treatment, resulting in the improvement of diarrhea [7].

In our patient, stopping this medication was not a viable option since it was very effective in reducing disease progression. Instead, we used budesonide, a glucocorticoid with high first-pass metabolism, as its systemic side effects would be less severe as compared with conventional glucocorticoids [7]. The patient had complete clinical remission in less than three months.

## Conclusions

Diarrhea and colitis are well-known common side effects of nintedanib and often lead to the discontinuation of this medication. In patients with nintedanib-induced colitis/diarrhea who are resistant to oral antidiarrheal medications, budesonide could be a viable option to cure this common side effect. Further research is needed to help standardize its use and prevent IPF treatment interruption.
